# Abstract of the Proceedings of the Buffalo Medical Association

**Published:** 1855-05

**Authors:** Sanford B. Hunt

**Affiliations:** Secretary


March, 1855.

---

ART. II.—*Abstract of the Proceedings of the Buffalo Medical Association.*

[These proceedings, which have occasionally been published in the "Journal," will hereafter appear regularly each month, in the form of an abstract of the more important items. The association is regularly and largely attended. Organized in 1845, it is now closing the first decade of its existence. Its importance is now fully recognized, and we are glad to notice that its membership is sought as a valuable privilege by all earnest minds in the profession of the city. The meetings are uniformly interesting, and we believe that in offering to our readers a regular report of its transactions, we shall present to them matter of the highest value in a condensed, readable, and authentic form. The present paper is intended to bring up to the present time whatever cases of interest remain yet unpublished. It accordingly runs back for a period of several months. In our June number (the first of vol. xi.) we shall give from the minutes a report of a remarkable case of Cyanosis, illustrated by engravings.—Ed. Buff. Med. Jour.]

*Dec.* 1854. *Motion occurring early in fœtal life.* Dr. Baker stated that his partner, Dr. T. T. Lockwood, recently brought to the office a human ovum, on account of its beauty, the fœtus lying enclosed in unbroken membranes which were perfectly transparent. It was brought some distance enveloped in a cloth, and lay for a little while upon a table in a cold room. The whole sac was about the size of a turkey's egg. From the history given by the mother she was about six weeks advanced in pregnancy. On examining it, Dr. Baker saw the fœtus first draw up one leg, and afterward move both arms and legs distinctly, with a quick, sharp, motion. This was done while the fœtus was lying undisturbed upon the table. Drs. Lockwood, King, and Newell, also saw it in motion, about an hour and a half after the time of its expulsion. Dr. White was asked to see it. He did so about 4,

P. M., two and a half to three hours after its expulsion, and saw very active motion through the membranes, which were beautifully clear.

This case is important as showing the incorrectness of the notion that quickening never occurs till the middle of the fifth month, and also in a medico-legal point of view, with reference to the criminality of procured abortions before quickening.

*Jan.*, 1855. *Blistering of Infants. Prevention of Strangury. Smith's "Tela Vesicatoria."* Dr. Root related a case of pneumonia in an infant, in which, among other remedies, he had made use of blistering. In a recent conversation a medical friend had denied the propriety of blistering an infant. Dr. R. inquired the sentiment of members relative to the use of blisters in infants, especially in pneumonia.

Dr. White mentioned a case of double pneumonia in an infant, where blistering seemed productive of much benefit. He was in the habit of using them upon children, with proper precautions, and had never seen any injury resulting.

A general discussion followed. It was the general opinion that blistering on infants was justifiable, but that the blister should be removed before filling, and that bibulous paper should be interposed between it and the skin, to prevent the absorption of the cantharides.

Dr. Nelson stated his preference for Smith's "Tela Vesicatoria," manufactured in Edinburgh and not yet introduced into this country. It differs in appearance from the common yellow blistering tissue, so largely in use, is much more certain in its action, and productive of less pain and inconvenience.

Dr. Hunt mentioned a means of preventing strangury from the use of the ordinary emp. cantharidis. The surface of the blister should be freely dusted over with dry powdered camphor gum. He had never known a blister thus prepared to cause strangury, even in patients ordinarily subject to it, and he had seen it very largely used.

*March*, 1855. *Interparietal Bone. Bright's Disease. Intussusception. Tuberculosis. Ossification of Mitral Valve.* Dr. Rochester presented a skull having an interparietal bone, such as is described by Von Tschudi, of Vienna, and other ethnologists, as being peculiar to some families of the ancient Peruvians, and quoted as supporting the doctrine of a diversity of human races. This skull is certainly not Peruvian, being purchased a little while since in New York, evidently fresh, and beautifully bleached. The bone in question was an equilateral triangle, occupying the angle between the parietals posteriorly, looking like a segment of the vertical portion of the occipital bone. Its sides measured 1$\frac{3}{4}$ inches each. It was agreed that, in this case at least, it was only a variety of the ossa Womiana. The saggittal suture was continued down to the ossa nasi.

The following pathological specimens were also exhibited by Dr. Rochester:

1st. A case of intussusception of the small intestine taken from an infant dying of phthisis. The intestine was returned upon itself for a distance of nearly two inches, and its calibre very much narrowed, but not to entire strangulation. Its existence was unsuspected before death.

2d. The lungs of the same patient. They presented a tubercular cavity in the apex of each lung, with well marked lobular pneumonia at the base.

3d. Two kidneys, one of them presenting the mottled appearance of Bright's disease in all its parts, and of a little more than the normal size. The other, taken from the same patient, was entirely atrophied. That this was really atrophy, and not a congenital malformation, was proved by the fact that the ureters and pelvis of the kidney were of the normal size. The parenchyma of the organ was, however, so wasted that it was only about one inch in length, and proportionally wide. No vestiges of the intimate structure of the organ could be found, only areolar tissue remained within the fibrous coat.

4th. Ossification of mitral valve. The patient affording this specimen had acute rheumatism at the age of 5 years, and repeated attacks during the remainder of his life. A year ago last fall he was admitted to the Hospital of the Sisters of Charity, where he had remained since, suffering from general anasarca. His diseased condition had so retarded his development, that he presented the appearance of a boy of 15, though he was well known to be 23 years of age. After his death, which occurred recently, a large ossification was found at the mitral valve. The body of the bony deposit occupied the place of the auriculo-ventricular rings at the base of the mitral valve, for an inch or more; and threw out bony excrescences upon the free surface of the valve, so as to very much diminish the orifice, while it prevented its entire closure at the moment of systole.

*April*, 1855. *Hydrophobia*. Dr. Hawley read the following report of cases of hydrophobia:

"Laurence Mier, German, aged 34 years, was bitten by his own dog, in the latter part of July, 1854, under the following circumstances: He had beaten the dog severely upon the head with a stick and then tied him to a tree.

Three or four days afterward, he untied the animal, giving him at the same time a kick, upon which the dog flew upon and bit his master's hand. The dog lived three days longer, refusing food and drink and snapping at all who approached him.

*Sept.* 14. Seven weeks after being bitten Mier became ill and complained to his wife of his throat. He took neither dinner nor supper that day, but performed his duty as watchman in the bell tower at night.

On Friday (Sept. 15) took no food, drank with difficulty, and suffered much from heat in the epigastrium, headache and diarrhœa. Took a half pint of brandy in addition to his usual pint of whisky. At night went to the tower and remained all night, but suffered intensely from thirst, heat in the epigastric region, and vomiting.

Saturday (Sept. 16) suffered in the same manner, and thought he would be happy if he could take a swallow of water; drank no spirits, but a little water with great difficulty. Went to the tower again at night but was unable to remain. On his return home called at my office, when I saw him for the first time. He was agitated and covered with cold perspiration; his pulse small and rapid; complained of intense thirst and a sense of burning at the epigastrium, and inability to drink on account of suffocation.

I presented him a little water, which he put to his lips with every expression of dread and hesitation, and swallowed convulsively, produced strangulation and universal agitation of the system. At this time he had no suspicion of hydrophobia, and said he had not been bitten by a dog in twenty years. I gave him a quarter grain of morphine and another to take when he reached home, and ordered free use of ice if he could take it. At one o'clock the next morning was called to see him, when he informed me that he remembered having been bitten by his own dog some weeks previous, and related the circumstances as above stated. He had eaten ice almost constantly with perfect relief to the gastric distress and great mitigation of the thirst. His aspect was still wild and tremulous, and his manner agitated.

*Sept.* 17. Saw him at seven o'clock. Appearance much the same; pulse about 120. Found his only relief in the use of ice. At 11 o'clock Dr. Eastman saw him with me, and corroborated the diagnosis. At 3 o'clock, P. M., Drs. White, Flint, Eastman, and Rochester met in consultation, confirmed the diagnosis and agreed to the performance of tracheotomy according to the recommendation of Dr. Marshall Hall. The operation was accordingly performed, and Dr. Hall's wire tube inserted. About three hours after the insertion of the tube, I was summoned in great haste, and found profuse hæmorrhage from the incision, removed the tube and arrested the bleeding by the application of ice. The hæmorrhage seemed to have been produced by his violent efforts to expel the coagula which gathered about the loops of wire and threatened suffocation. He passed the night in comparative quiet, and ate ice and small pieces of bread, the first food he had taken since Thursday morning. The drinking of fluids was attended with less difficulty on account of the admission of air through the opening in the trachea while the glottis was closed.

*Monday, Sept.* 18. In the morning a common tracheal tube was introduced with the belief that it would irritate the trachea less than the wire. But by no device was I able to retain it long in its place. All attempts at keeping the tube in the aperture were then abandoned. In the early part of this day, delirium, resembling that of mania a potu, came on and greatly complicated the hydrophobic symptoms.

In consequence of his delirious threatenings of his attendants, he was lashed to his bed. In this condition he remained suffering from delirium and spasms of the respiratory organs, till about midnight, when he fell into a stupor and died.

Up to Monday morning he retained full possession of his mental faculties, and was mild and harmless in his behaviour.

On the 19th of January, 1854, was called to see James Coho, a young vigorous Irishman. He had severe headache, intense thirst, and a small rapid pulse. The slightest motion of the head or of the bed clothes near his face, produced a catching, gasping respiration, precisely similar to that of a person who is unexpectedly plunged into cold water. I presented a teaspoonful of solution of morphine, which produced the suffocation, respiration, and universal shudder. He declared he had never been bitten by a dog, and I refrained from communicating to him my fears that he had hydrophobia. The next day visited him several times and persuaded him to attempt to take remedies in a fluid form. When after much reluctance he consented to try, he would stand upon the floor, with a strong man upon each side holding him firmly, with his eyes glaring and every muscle strained as if for desperate effort, he would throw the fluid down his throat and leap to the window and thrust his body far out into the air for breath. His behaviour after an attempt to swallow a fluid, resembled that of a person in a violent fit of spasmodic asthma.

The next day his conduct became so violent that it was not deemed safe to keep him longer at home, and he was sent to the county poorhouse, where he died on the following day.

I have added this case because it agrees with the other in that the patient had no suspicion, and consequently no fear of the disease, and yet afforded unequivocal signs of hydrophobia."

Dr. Rochester stated in this connection, that last year a letter was handed to him by the editor of the Commercial Advertiser of this city, with the request that he would answer it. It proved to be from a gentleman in New Jersey, one of whose employees had been bitten by a rabid dog. Hearing of hydrophobia in Buffalo, the gentleman had addressed this letter asking advice. Dr. R. answered recommending excision of the wound. A few days since he received a letter from the gentleman, stating that the wound was excised ten days after the bite, that six months had now elapsed, and the man remained entirely well.

Dr. Newman stated that early in 1854 a boy was reported to the Board of Health, as dying of hydrophobia, by Drs. Weiss and Nichols. Inquiry was made into the circumstances, and it was found that the boy died with all the symptoms of hydrophobia, but that the dog that inflicted the bite manifested no symptoms of disease. The father of the boy kept this dog some three or four weeks after his son's death, without observing any signs of rabies, and finally killed him to be rid of him.

*Pneumonia.* Dr. Strong described a case of pneumonia, attended by subacute pleurisy with considerable serous effusion. The peculiarity of the case was that the patient had very little pain or cough, and no expectoration, and was so little affected as to keep about his business until hepatization had taken place.

Dr. Hunt mentioned four similar cases, in which bloody sputa were the only occasion of his being called in. All did well with a very trivial treatment. He regarded them as evidence of the self-limited nature of pneumonia.

SANFORD B. HUNT, M. D., Secretary.

